# The mineralocorticoid receptor is essential for stress axis regulation in zebrafish larvae

**DOI:** 10.1038/s41598-018-36681-w

**Published:** 2018-12-27

**Authors:** Erin Faught, Mathilakath M. Vijayan

**Affiliations:** Department of Biological Sciences, University of Calgary, 2500 University Drive NW, Calgary, Alberta T2N 1N4 Canada

## Abstract

The mineralocorticoid receptor (MR) in mammals mediates the effects of aldosterone in regulating fluid balance and potassium homeostasis. While MR signalling is essential for survival in mammals, there is no evidence that MR has any physiological role in ray-finned fish. Teleosts lack aldosterone and emerging evidence suggest that cortisol mediates ion and fluid regulation by activating glucocorticoid receptor (GR) signalling. Consequently, a physiological role for MR signalling, despite its conserved and ancient origin, is still lacking. We tested the hypothesis that a key physiological role for MR signalling in fish is the regulation of stress axis activation and function. Using either MR or GR knockout zebrafish, our results reveal distinct and complementary role for these receptors in stress axis function. GR^−/−^ mutants were hypercortisolemic and failed to elicit a cortisol stress response, while MR^−/−^ mutants showed a delayed, but sustained cortisol response post-stressor. Both these receptors are involved in stress-related behaviour, as the loss of either receptors abolished the glucocorticoid-mediated larval hyperactivity to a light stimulus. Overall, the results underscore a key physiological role for MR signalling in ray-finned fishes, and we propose that the regulation of the highly conserved stress axis as the original function of this receptor.

## Introduction

The primary role of the mineralocorticoid receptor (MR) in mammals is to mediate the effects of aldosterone in regulating fluid balance and potassium homeostasis. It is essential for survival in mammals as MR-null mice die 1–2 weeks postnatally from renal salt wasting and hyperkaliemia^1^. The physiological importance of MR is evident from its persistence in vertebrate evolution. MR exists in every major vertebrate clade, and even the agnathans have a corticosteroid receptor (CR), which is thought to be an ancestral form of MR, while the glucocorticoid receptor (GR) ortholog is first seen in the elasmobranch lineage^2^. Despite its persistence, there is no known physiological role for MR in ray-finned fish^3^. Also, teleosts lack aldosterone, and glucocorticoid appears to mediate most of the changes in iono- and osmo-regulatory functions by activating GR signalling^4^.

In mammals, in addition to aldosterone, the glucocorticoids are also a major ligand of MR; however, almost all the mineralocorticoid functions are mediated by aldosterone-MR signalling^5^. The high affinity of cortisol for MR means that aldosterone-mediated effects occur in tissues where 11β-hydroxysteroid dehydrogenase 2 (11β-HSD2), a key enzyme that breaks down cortisol, is prevalent. Tissues that lack 11β-HSD2, but still contain MR, suggests that this receptor also has an extra-mineralocorticoid role in mammals and activated by glucocorticoids^6,7^. Fish MR can be transcriptionally activated by several 3-ketosteroid hormones, including cortisol, 11-deoxycortisol, corticosterone and 11-deoxycorticosterone, suggesting its possible physiological significance^3^. Cortisol is the primary glucocorticoid in teleosts and this hormone is released during stress to promote energy substrate mobilization and metabolic recovery post-stressor^8,9^.

Vertebrates share a highly conserved corticosteroid stress response that is central to stress adaptation^8^. As in mammals, activation of the hypothalamus-pituitary-interrneal (HPI; analogous to the HPA) axis in fish commences with the release of corticotropin-releasing hormone (CRH) from the hypothalamus, and this peptide stimulates the release of adrenocorticotropic hormone (ACTH) from the pituitary into the circulation^8,10^. ACTH binds to the melanocortin 2 receptor (MC2R) on the interrenal cells (analogous to the adrenal cortex in mammals), distributed in the head kidney region, stimulating the biosynthesis of cortisol in teleosts^11^. HPI axis activation by stressors elevates circulating cortisol levels, which facilitates glucose mobilization to increase energy availability in target tissues and restore homeostasis. The cortisol-driven molecular programming of HPI axis development has focused only on GR signalling in fishes^12–17^. Although an MR knockout was generated in medaka fish (*Oryzias latipes*), a role for this receptor in HPI axis functioning was not addressed^18^. In mammals, the ratio of GR: MR signalling is thought to play a role in the stress-related behaviour^19^, and the MR function may be brain region-specific^20^. Similarly, loss of MR in medaka also altered adult behaviour, suggesting a conserved role for MR in regulating stress-related behaviour^18^.

To determine a physiological role for MR signalling in teleosts, and given the ancient origin of this receptor, we tested the hypothesis that MR signalling regulates the highly conserved stress axis function. To this end, we generated ubiquitous MR^−/−^ and GR^−/−^ knockouts in zebrafish (*Danio rerio*) using CRISPR/Cas9 mutagenesis. Zebrafish is an ideal model for loss-of-function studies because, unlike other teleosts with paralogs for GR^21^, it only has a single MR and GR in the genome. Our results for the first time highlight a key physiological role for MR signalling in fish.

## Results

### Generating MR and GR knockouts in zebrafish

**Figure 1:**
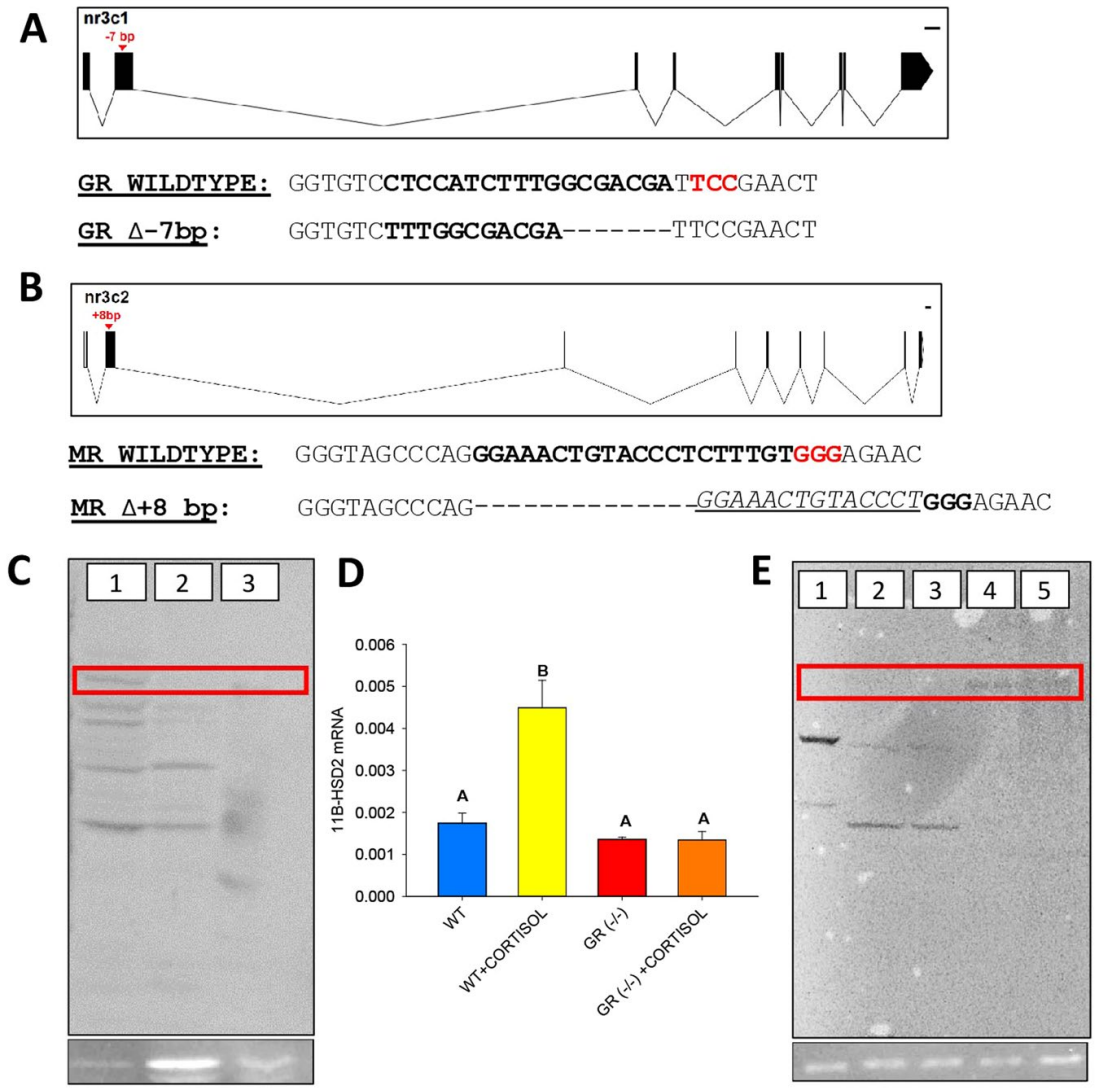
GR and MR knockout in zebrafish: (**A**) Schematic representation of the zebrafish GR (nr3c1) gene. Exons are shown in boxes, introns are denoted by lines. The arrow shows the position of a −7 bp deletion in exon 2. Detailed below the schematic, the protospacer-adjacent motif (PAM) is denoted in red and the target site is bolded. (**B**) Schematic representation of the zebrafish MR (nr3c2) gene. Exons are shown in boxes, introns are denoted by lines. The arrow shows the position of a + 8 bp deletion in exon 2. Detailed below the schematic, the protospacer-adjacent motif (PAM) is denoted in red and the target site is bolded. (**C**) Western blotting with anti-trout GR (1:1000). Lane 1) WT zebrafish whole-body; Lane 2) GR^−/−^ zebrafish whole-body; Lane 3) WT trout liver. Red box denotes area of interest corresponding to GR protein at ~90 kDa. (**D**) Transcript abundance of 11β-HSD2; bars are wildtype [WT], wildtype treated with cortisol [WT + Cortisol], GR knockout [GR(−/−)], GR knockout treated with cortisol [GR(−/−) + Cortisol]; bars with different letters are significantly different (One-way ANOVA, p < 0.05, n = 4, bars are mean ± SEM). (**E**) Western blotting with anti-zebrafish MR (1:500). Lane 1) WT trout liver; Lane 2) WT zebrafish liver; Lane 3) MR^−/−^ zebrafish liver; Lane 4) WT zebrafish head; Lane 5) MR^−/−^ zebrafish head. Red box denotes area of interest corresponding to MR protein at ~110 kDa. Representative image below each blot shows β-actin expression as a loading control.

To determine the roles of GR and MR in HPI axis programming and the development of stress-related behaviour, we generated homozygous GR^−/−^ and MR^−/−^ zebrafish mutants^22^. The GR^−/−^ mutants had an 18 bp deletion and an 11 bp insertion (net −7 bp change) in exon 2 (n-terminal domain of the GR protein), which was 232 bp downstream of the start codon in gene nr3c1 (Fig. 1A). *In silico* analysis predicted that a premature stop codon at ~500 bp downstream of the start codon would result in a severely truncated protein. This was confirmed by western blotting (Fig. 1C), and the loss of function was indicated by the abolishment of the glucocorticoid-induced elevation in 11β-HSD2 mRNA levels (Fig. 1D). The MR^−/−^ mutants had −5 bp deletion and a +13 bp insertion (net +8 bp change) in exon 2, which was 414 bp downstream of the start codon in gene nr3c2 (Fig. 1B). This corresponds to amino acid position 140, which is in the n-terminal domain of the protein. *In silico* analysis predicted that a premature stop codon should be encountered ~70 bp downstream of the NGG site, resulting in a truncated protein ~500 bp downstream of the start codon. MR^−/−^ knockout was confirmed by Western blotting that showed the absence of MR protein expression (Fig. 1E), but a MR-specific gene target is yet to be reported in teleosts.

### MR larvae have normal cortisol levels, whereas GR larvae are hypercortisolemic

**Figure 2:**
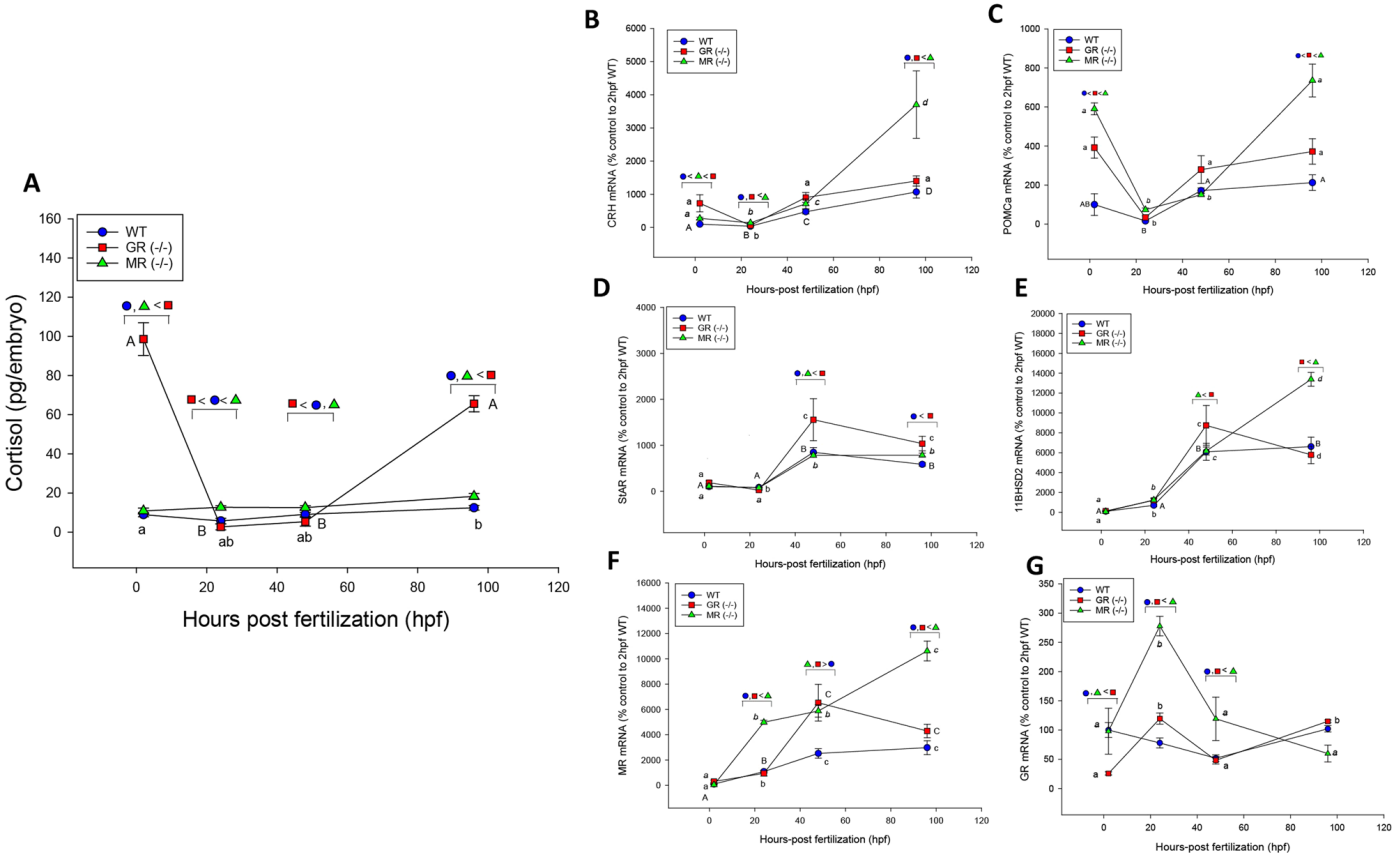
GR and MR regulate genes involved in hypothalamus-pituitary-interrenal (HPI) axis activity during development: Ontogeny of the cortisol response (**A**) and the transcript abundance of HPI axis-related genes (**B**–**G**) in the wildtype (WT), GR knockout [GR(−/−)] and MR knockout [MR(−/−)] embryos, and transcript abundance of HPI axis intermediates at 2, 24, 48, 96 hpf. (**B**) Corticotropin-releasing hormone (CRH), (**C**) Proopiomelanocortin a (POMCa), the gene for the precursor protein to ACTH, (**D**) Steroidogenic acute regulatory protein (StAR), a rate-limiting step in steroid hormone biosynthesis, (**E**) 11β-hydroxysteroid dehydrogenase (11β-HSD2), which catalyzes the breakdown of cortisol to biologically inactive cortisone. (**F**) Mineralocorticoid receptor (MR) and the glucocorticoid receptor (GR). A significant interaction was detected in all two-way ANOVAs, p < 0.05. Significant time effects within treatment groups are indicated by different letters (WT: A, GR−/−: a, MR−/−: a); significant treatment effects within each time points are indicated above each time point using legend symbols. All data points are mean ± SEM (n = 4–6; each n is pool of 10 larvae).

Here we report distinct changes to the HPI axis development as a result of a GR or MR knockout in zebrafish (Fig. 2). While MR^−/−^ embryos showed cortisol levels comparable to that of the WT over the sampling period, the cortisol levels in the GR^−/−^ mutants were exceptionally high at 2 and 96 hpf compared to the other two groups (Fig. 2A). High cortisol levels at 2 hpf in GR^−/−^ reflects the maternal cortisol levels that were transferred to the embryo^23^. There was a drastic drop (p < 0.001) in the GR^−/−^ embryo cortisol content (2.7 ± 1.5 pg/embryo) at 24 hpf compared to 2 hpf, and this level was significantly lower than the WT at 24 (p = 0.050) and 48 hpf (p = 0.036). The MR^−/−^ embryos showed no decrease in cortisol levels at 24 hpf (12.70 ± 0.82 pg/embryo), and they had 2-fold higher cortisol levels compared to both GR^−/−^ (p < 0.001) and WT (p = 0.013) at this time-point (Fig. 2A).

### MR regulates genes involved in HPA axis activity during development

To determine the possible mechanisms behind the altered cortisol profile, we measured the transcript abundance of key HPI axis genes. There were distinct changes in the transcript abundances of key genes in response to either GR or MR loss (Fig. 2B–G).

*CRH*: Both GR^−/−^ and MR^−/−^ mutants have increased *crh* transcript abundance at 2 hpf compared to WT (p < 0.001 and p = 0.003, respectively), with GR^−/−^ embryos having a greater abundance compared to MR^−/−^ (p = 0.031). At 24 hpf, loss of MR resulted in a greater *crh* mRNA abundance compared to WT embryos (p < 0.001) and GR^−/−^ embryos (p = 0.009). At 24 hpf, the *crh* mRNA levels were similar in the GR^−/−^ and WT (p = 0.158). At 48 hpf, there was no difference in the transcript abundance between the groups, but at 96 hpf, *crh* transcript level was 4-fold higher in the MR^−/−^ compared to WT (p < 0.001) and GR^−/−^ (p < 0.005) (Fig. 2B).

*ACTH*: Proopiomelanocortin (POMC) is encoded by the *pomca* gene in zebrafish and is the precursor protein for ACTH, which is released upon CRH stimulation^21^. WT *pomca* transcript abundance increases over the first 120 hpf in zebrafish (Fig. 2C). At 2 hpf, both GR^−/−^ and MR^−/−^ mutants have higher *pomca* transcript abundance compared to WT (p < 0.001), with MR^−/−^ having a greater abundance compared to GR^−/−^ (p = 0.028). There was no difference at 24 hpf between any of the three groups as *pomca* mRNA levels decreased from 2 hpf in both the GR^−/−^ (p < 0.001) and MR^−/−^ embryos (p < 0.001). At 48 hpf, all embryos had similar transcript levels. However, by 96 hpf, *pomca* abundance in GR^−/−^ larvae is greater than WT larvae (p = 0.024), while MR^−/−^ larvae had increased significantly from its levels at 48 hpf (p < 0.001) and the transcript abundance was greater than both WT (p < 0.001) and GR^−/−^ larvae (p < 0.011) (Fig. 2C).

*StAR*: A key rate-limiting step in corticosteroid biosynthesis is the StAR protein, which shuttles cholesterol to the inner mitochondrial membrane^24^. During early development, *star* transcript abundance increases after hatch in zebrafish^21^ (Fig. 2D). The maternal deposition of *star* transcripts was not affected by the loss of GR or MR compared to the WT. Similarly, there was no difference between the different treatments at 24 hpf, while at 48 hpf all groups had increased *star* mRNA levels compared to 24 hpf (p < 0.001), with GR^−/−^ larvae having greater abundance compared to WT (p = 0.009) and MR^−/−^ larvae (p = 0.010) (Fig. 2D).

*11β-HSD2*: Another key player in modulating cortisol levels during early development is the enzyme 11β-HSD2 that break down cortisol to its inactive form cortisone for elimination^25^. As reported previously, *11β-hsd2* transcript levels are deposited in low amounts from the mother and increase by hatch (48 hpf; Fig. 2E)^21^. While there was no difference in *11β*-*hsd2* transcript abundance prior to hatch, in the mutants, there was a clear receptor-specific effect on this transcript profile after hatching in zebrafish (Fig. 2E). At 48 hpf, GR^−/−^ have significantly higher *11β-hsd2* compared to MR^-/-^ (p < 0.001), but not WT larvae (0.061). Transcript abundance of *11β-hsd2* in MR^−/−^ continue to increase at a greater rate compared to either WT or GR^-/-^ larvae, and by 96 hpf, MR^−/−^ have greater *11β*-*hsd2* transcript abundance compared to GR^−/−^ larvae (p = 0.005). GR^-/-^ and WT have similar levels of *11β-hsd2* mRNA at 96 hpf (p = 0.082) (Fig. 2E).

### GR and MR knockouts alter the developmental profiles of their respective receptors

To test whether the dysregulation of cortisol levels during early development are intricately linked to GR and MR signalling, we also characterized the ontogeny of *gr* and *mr* transcript abundance in the mutants during early development (Fig. 2F–G). It should be noted that while the *mr* and *gr* are present in the respective knockouts, these are not indicative of protein levels (Fig. 1B,E).

*MR*: In the WT, *mr* is deposited in low amounts and steadily increases over the first 4 days of zebrafish development^21^ (Fig. 2F). A loss of MR caused an increased amount of *mr* mRNA at 24 hpf compared to both WT (p < 0.001) and GR^-/-^ embryos (p < 0.001). GR^-/-^ and MR^-/-^ mutants had similar mRNA levels of *mr* at 48 hpf (p = 0.672), which is significantly more than WT embryos (p < 0.001). At 96 hpf, *mr* mRNA levels were greatest in the MR^-/-^ larvae compared to the GR^-/-^ (p < 0.001) and WT larvae (p < 0.001) (Fig. 2F).

*GR*: *gr* transcript abundance in WT embryos decreases until hatch, after which the mRNA levels increase (Fig. 2G). A loss of GR caused substantially fewer *gr* transcripts to be deposited into the embryos compared to WT (p = 0.002) and MR^-/-^ (p = 0.006). A loss of either receptor caused an increase in *gr* at 24 hpf from 2 hpf (p < 0.001), with MR^-/-^ having a greater amount of *gr* mRNA compared to WT and GR^-/-^ embryos (p < 0.001). At 48 hpf, there was no difference between GR^-/-^ and WT in *gr* mRNA levels; however, the *gr* mRNA levels in MR^-/-^ was still greater than both GR^-/-^ (p = 0.024) and WT (p = 0.028). All larvae had similar *gr* transcript abundance at 96 hpf.

### GR and MR differentially regulate the glucocorticoid stress response

**Figure 3:**
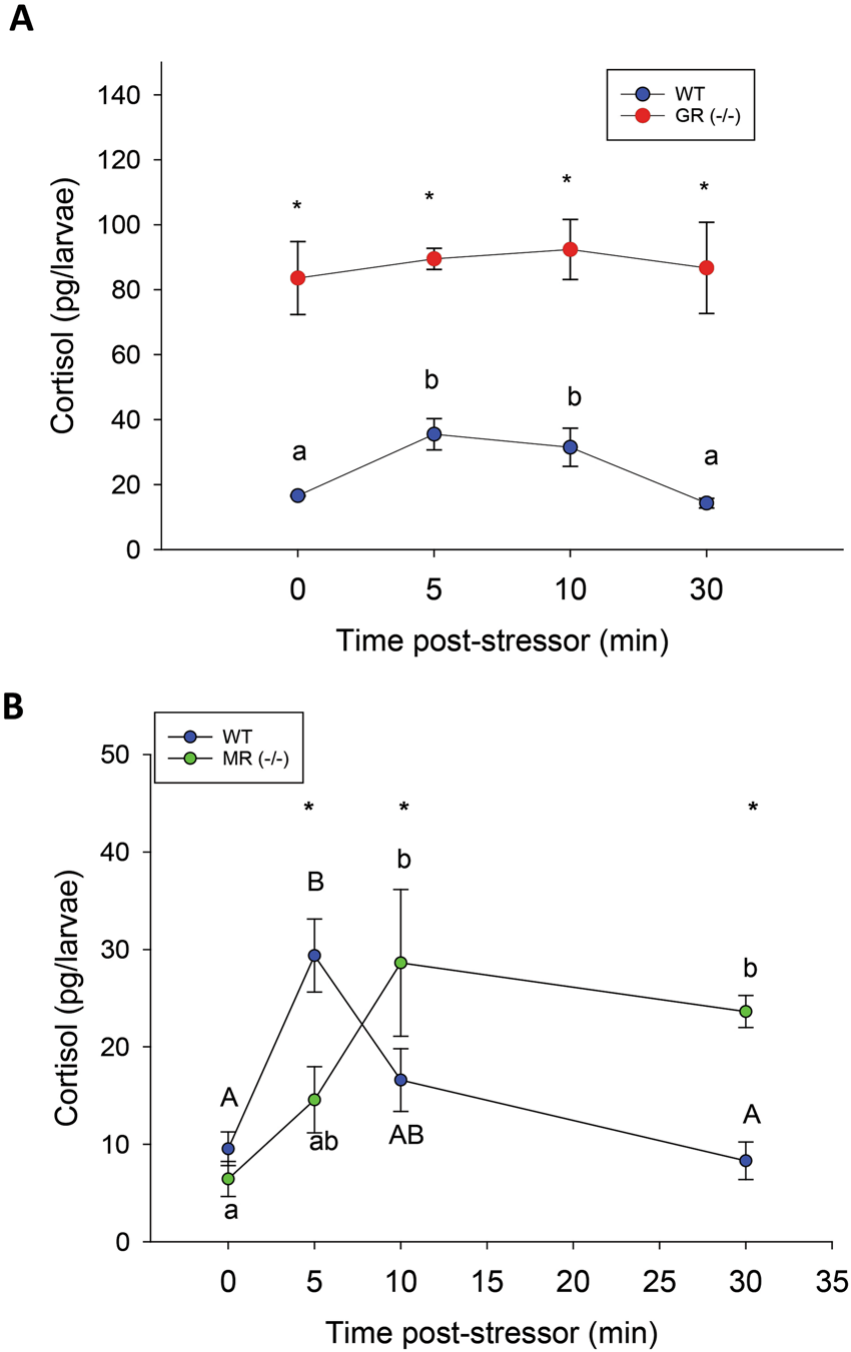
GR and MR are necessary for the cortisol profile post-stress: (**A**) Loss of GR [GR(−/−)] results in hypercortisolemic larvae at 96 hpf, which did not respond to a swirling (250 rpm) stressor. Their cortisol levels are beyond what could be induced in the wildtype [WT] larvae. B) Loss of MR [MR(−/−)] causes a delay in the timing of peak cortisol levels compared to WT at 96 hpf, but does not change the magnitude of the stress response. MR^−/−^ larvae are unable to restore cortisol levels to the resting level. A significant interaction was detected in both two-way ANOVAs, p < 0.05. Significant time effects within treatment groups (WT uppercase and knockout lowercase) are indicated by different letters; Significant treatment effects within each time-period is indicated by an asterisk. All data points are mean ± SEM (n = 4–6; each n is a pool of 10–15 larvae).

To determine the integrity of the HPI axis and its capacity to elicit a cortisol stress response in the mutants, we subjected the larvae to an acute stressor and measured larval cortisol levels post-stressor exposure^21^. As expected, WT basal cortisol levels (16.6 ± 0.2 pg/larva) doubled to 35.5 ± 4.8 pg/larva at 5 min (p = 0.007) and returned to resting levels at 30 min (p = 0.468) post-stressor exposure (Fig. 3A,B). The GR^−/−^ larvae did not elicit a cortisol stress response and the steroid levels were consistently ~4-fold higher than the WT at all time points (p < 0.001) post-stressor exposure (Fig. 3A). The MR^−/−^ larvae were able to respond to an acute stressor by elevating cortisol levels, but there was a delay in HPI axis activation compared to the WT (Fig. 3B). There was no difference in the basal cortisol levels between WT and MR^−/−^ prior to stress (p = 0.554), or in the magnitude of the stress response post-stress compared to the WT. However, the loss of MR led to a delay (5 min) in the attendant rise in cortisol post-stress as MR^−/−^ larvae experienced peak cortisol levels only at 10 min post-stressor exposure (p = 0.003; Fig. 3B). Also, unlike the WT, the cortisol levels did not return to resting levels by 30 min (p = 0.020) in the MR^−/−^ larvae (Fig. 3B).

### Both GR and MR are required for stress-related larval behaviour

**Figure 4:**
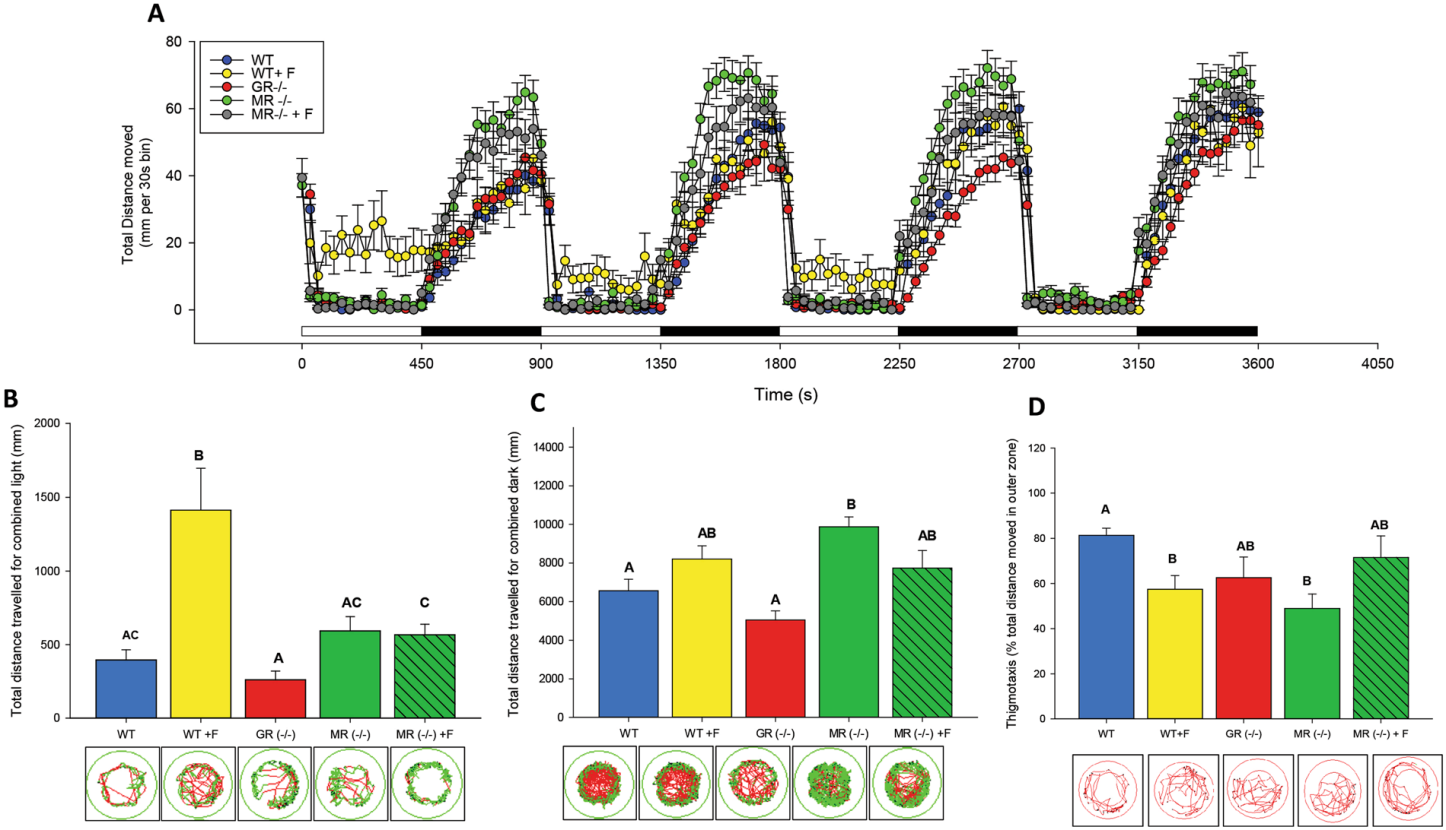
GR and MR are necessary to mediate stress-related behaviour: (**A**) Behavioural profiles of 96 hpf zebrafish larvae that were either wildtype [WT], wildtype treated with cortisol [WT + F], GR knockout [GR(−/−)], MR knockout [MR(−/−)] or MR knockout treated with cortisol [MR(−/−) + F]. Mean activity in light and dark is represented, where the activity of 96 hpf larvae is expressed as total distance moved during each 30 s recording bin. The total recording period was 1 h with alternating light periods of 7.5 min each. (**B**) Total combined distance moved (mm) in the light and (**C**) total combined distance moved in the dark period. Representative tracking data is included under each bar. Bars represent mean ± SEM (n = 24). Different letters indicate significant differences (One-way ANOVA, p < 0.05). (**D**) Thigmotaxis: Arenas (wells) were divided into inner and outer zones, and the propensity of 96 hpf larvae to stay close to the outer arena wall was expressed as % of total distance travelled (inner and outer arena) in 30 min. Bars represent mean ± SEM (n = 12). Different letters indicate significant differences. Representative paths for thigmotaxis are shown below each bar.

Given the differences in the physiological stress response, we also evaluated whether the stress-related behaviour was altered in the CR mutants. Zebrafish have become an increasingly powerful model organism for translational neuroscience research^26^, and glucocorticoids are known to cause hyperactivity in larvae^27,28^. To determine if CRs are involved in this stress-induced larval behaviour, we subjected the larvae to a light/dark stimuli^28^ (Fig. 4A). As shown previously, larvae treated with cortisol showed a higher activity in the light compared to the WT (Fig. 4B), but there was no difference in locomotor activity in the dark^28^ (Fig. 4C). This cortisol-induced behavioural response was completely abolished in the GR^−/−^ larvae, despite being hypercortisolemic (Fig. 4B). MR^−/−^ larvae did not show this hyperactive response in the light (Fig. 4A). However, as MR mutants have cortisol levels similar to that of the WT, we treated MR^−/−^ larvae with cortisol to determine whether cortisol-induced hyperactivity was GR-mediated. Interestingly, even with cortisol, we were not able to recover the cortisol-induced hyperactivity (Fig. 4A). To determine whether this increase in activity was a reflection of increased boldness (decreased anxiety), we also looked at thigmotaxis, or the tendency to remain close to the periphery of the arena. This is used as an index of anxiety in both mice^29^ and zebrafish^30^. As previously reported fish treated with cortisol have decreased thigmotaxis (p = 0.027), displaying an increased boldness^28^ (Fig. 4D). A loss of MR also caused the larvae to be bolder, compared to WT (p = 0.045), suggesting that MR may be a primary mediator of anxiety behaviour. However, treatment with cortisol reduced the boldness of the MR^−/−^ larvae to that of WT, suggesting GR may also be necessary for anxiety-related behaviour as a loss of GR caused a partial recovery to baseline WT activity (80% of total time in the periphery) (Fig. 4D).

## Discussion

While the role of GR is well defined in teleosts, nothing is known about the physiological role directly mediated by MR signalling. Here we report that MR is also a key player in affecting the stress response of lower vertebrates. By comparing CR knockout zebrafish lines, we reveal distinct and complementary roles for MR and GR signalling in the development of the stress axis. The results indicate that both GR and MR signalling are involved in the negative feedback regulation of cortisol during stress. GR is essential for maintaining the steady-state resting levels of glucocorticoids, whereas MR is involved in both the perception of stress and for cortisol homeostasis after an acute stressor (Fig. 3A,B). Our results also suggest a role for MR in the repression of key genes involved in the HPI axis function^10^, while the role of GR may include the regulation of cortisol metabolism (upregulation of 11β-HSD2). The distinct roles of GR and MR in regulating HPI axis programming are particularly important given that dysregulated cortisol levels during embryogenesis lead to developmental defects in fish, including impaired HPI axis activity later in life^31–34^. Furthermore, both receptors mediate cortisol-induced behaviour in larvae indicating that CRs play a central role in behavioural outcomes in fish (Fig. 4A–D). Although this is well established in mammals^6,7^, a similar functional role of MR signalling in shaping stress-related behaviour in fish was lacking^18^. As behaviour is an essential component of the stress-coping mechanism^19^, a loss of either receptor would have maladaptive consequences to an organism’s ability to adapt to stress, and this may explain the persistence of MR and GR in the vertebrate phylogeny. Together, these results provide a strong physiological underpinning for the evolutionarily conserved role for MR in the regulation of stress axis function in vertebrates.

In fish, stress and the attendant rise in cortisol levels can be transferred to the developing oocytes, which can be detrimental to proper embryogenesis and development of the stress axis^23,32^. As such, cortisol deposited into the oocyte is tightly regulated by cortisol-mediated upregulation of 11β-HSD2, a key enzyme that inactivates cortisol to cortisone^23,35^. The exceptionally high cortisol levels in the GR^−/−^ mutants at 2 hpf reflect the transfer of this steroid from the hypercortisolemic mother^23^. This may be due to a lack of oocyte buffering by *11β-hsd2* in the GR^−/−^ mutants (Fig. 1D), as GR signalling upregulates this gene expression in teleosts^23,35^. The role of 11β-HSD2 in regulating oocyte cortisol levels is similar in function to the protective role this enzyme has in the developing fetus in mammals^25^, indicating a critical role for GR, but not MR in maintaining the steady-state cortisol levels in the embryos. While the dynamics of maternal contribution of hormones and transcripts were outside the scope of this paper, it would be an interesting avenue for future research to examine GR-regulated maternal depositions and their contributions to progeny development. This can be achieved by comparing the maternal-zygotic mutants that we have generated to the zygotic mutants, which would have partial maternal deposition from their heterozygous mothers.

Despite the high maternal cortisol deposition in GR^−/−^ embryos, this steroid is cleared rapidly, and the low cortisol levels are maintained for 24 h. While the mechanism is unclear as *11β-hsd2* transcript levels are low in GR^−/−^ larvae (Fig. 2A), the excretion of this excess steroid may involve either diffusion^36^, activation of other biotransformation pathways^37^ and/or upregulation of efflux transporters^38^. Except for this brief period, GR^−/−^, but not MR^−/−^mutants were hypercortisolemic and the steroid levels were beyond what could be induced by a stressor in the WT larvae (Fig. 2A)^14^. This suggests either a non-functional negative feedback mechanism and/or a lack of cortisol breakdown in the GR mutant. In contrast to GR, a loss of MR resulted in basal cortisol levels similar to WT (Fig. 2A), supporting a key role for GR signalling in maintaining the steady-state basal glucocorticoid levels during early development^32^, and this may involve a tight negative feedback regulation in fish^39^.

Our results indicate a key role for MR signalling in tightly regulating the molecular components of the HPI axis during early development. This is clearly evident from the lack of regulation of *crh* (Fig. 2B) and *pomca* (Fig. 2C) transcript levels during HPI axis ontogeny. Despite elevated *crh* and *pomca* transcript abundance in the MR^−/−^ mutants at 96 hpf, the larvae were not hypercortisolemic suggesting rapid cortisol clearance. Indeed, *11β-hsd2* is upregulated at this time point and may be a primary cause for maintaining basal cortisol levels (Fig. 2E). The control of HPI axis intermediates by MR is further supported by the lack of changes in these transcripts in the GR^−/−^ mutants, which also has a MR. These results clearly indicate that GR and MR have distinct roles in HPI axis function; MR repressing HPI axis activation during development, while GR regulates basal cortisol levels. Also, the delayed cortisol response, as well as a dysregulated cortisol level after an acute stressor in the MR^−/−^ mutants (Fig. 3B), suggests a physiological role for this receptor in regulating the stressor-mediated HPI axis activity. As vertebrates share a highly conserved corticosteroid stress response that is central to stress adaptation^8^, this study highlights the necessity of having both a functional MR and GR for HPI axis development and stress signalling.

In addition to HPI axis activity, the corticosteroid receptors are also involved in stress-related behavioural changes^14^. The cortisol-mediated activity of the larvae in the light and dark (Fig. 4A–C) were affected by the loss of GR or MR supporting a complementary role for both these receptors in stress-related behavioural outcomes. In addition, the lower thigmotaxis seen with cortisol in the wildtype was also seen in the MR^−/−^ mutant, suggesting that GR may be the primary mediator of this behaviour (Fig. 4D). However, the partial recovery to WT behaviour after exposure of MR^−/−^ to cortisol suggests that the response is not as simple as either the presence or absence of receptors, but involves a complex interaction of both receptors in mediating specific behavioural outcomes. Indeed, an often-overlooked aspect of CR signalling is the MR:GR ratio^24,27^, which may be involved in regulating emotional responses, including fear and anxiety-related behaviour^40^, and hyperactivity to novel stimuli^6^. Our results suggest a key role for GR and MR signalling in the behavioural responses mediated by stressor-mediated elevated cortisol levels. While the mechanism for receptor interaction in mediating these responses is unclear, one possibility is the GR and MR heterodimerization, and the resultant transactivation or transrepression of genes controlling behaviour^41^.

In conclusion, we report that a key physiological role for MR signalling in fish is to regulate stress axis function during early development. While it is currently unknown how a lack of corticosteroid signalling might impact the physiology of adult fish, perturbations in the glucocorticoid system in early life have been correlated with adult behavioural changes^42^. Furthermore, any changes in the cortisol stress response may cause fundamental alterations in intermediary metabolism as this is a primary target of corticosteroid signalling in adults^9^. We also highlight that the presence of GR alone is clearly not sufficient to mediate the physiological role of stress or stress-related behaviour in fish. As the glucocorticoid stress response is highly conserved in vertebrates and essential for stress adaptation^8^, we propose that stress axis regulation is a potential early role for MR signalling in vertebrates. While other physiological roles may exist, the MR regulation of the larval stress response in zebrafish provides a functional underpinning to the early origin of this receptor in vertebrates^3^.

## Materials and Methods

### Zebrafish maintenance

Adult zebrafish (Tupfel long fin (TL) strain) were maintained on a recirculating system with a 14:10 light: dark cycle (Pentair Aquatic Habitats, Florida, USA). All experimental protocols were approved by the Animal Care and Use Committee at the University of Calgary (AC17-0079), and were in accordance with the Canadian Council on Animal Care guidelines. Water was maintained at 28.5 °C, pH 7.6, and 750 µS conductivity, and 10% of the water was exchanged daily. Animals were fed twice daily with Gemma micro 300 diet (Skretting, USA) in the morning and live *Artemia* (San Francisco Bay Brand, USA) in the afternoon.

Zebrafish embryos/larvae were reared for days 0–5 dpf in a 28.5 °C incubator in 10 cm Petri dishes (Sarstedt, USA) at a density of 100 embryos/dish in E3 embryo media (5 mM NaCl, 0.17 mM KCl, 0.33 mM CaCl_2_, 0.33 mM MgSO_4_ + 0.1 ppm methylene blue antifungal agent^43^). Embryos/larvae were raised on a 14 h light: 10 h dark cycle, and 50% of the embryo media was replaced daily. Embryos/larvae were euthanized with MS222 (0.3 g/L) at 2, 24, 48, and 96 hpf for cortisol and transcript analysis and stored at −80 until use. Larvae that were being raised were fed starting at 5 dpf. Briefly, 5dpf larvae were transferred to a 3 L tank with 1.5 L of water and kept in temperature-controlled room at 28.5 °C on a 14 h light:10 h dark cycle. Larvae were fed AP100 larval food (Ziegler, USA) and Gemma micro 150 (Skretting, USA) in the morning, and live *Artemia* in the afternoon, and 50% of the water was changed daily until 15 dpf. At 15 dpf larvae were transferred to the recirculating system (Aquatic Habitats, Pentair, USA or Tecniplast, Italy) and maintained as described above.

### Generation of nr3c1 and nr3c2 null zebrafish

Nr3c1 and nr3c2 null fish were generated as exactly as described previously^22^. Briefly, a zebrafish specific Cas9 containing plasmid (Addgene plasmid #46757) was linearized by digestion with XbaI (New England Biolabs) for 2 h at 37 °C. Cas9 mRNA was immediately generated using *in vitro* transcription using the T3 message kit (Life Technologies) according to the manufacturer’s directions. sgRNA targets were designed (http://zifit.partners.org/ZiFiT/, or http://www.crisprscan.org/) to the second exon (first coding exon) of both nr3c1 and nr3c2 (Ensembl genome browser). Primers were also designed to create a 200–300 bp amplicon surrounding the target region. Target sequences (20 bp) were tagged with a 5’T7 sequence (TAATACGACTCACTATA) for *in vitro* transcription and a 3′ complementary sequence (GTTTTAGAGCTAGAAATAGC) to the universal ultramer. Both the sgRNA and the utltramer were ordered as DNA oligomers, annealed and extended using the following parameters: 98 °C, 2 min; 50 °C, 10 min; 72 °C, 10 min; 4 °C hold. The resulting product provided a template for sgRNA synthesis by *in vitro* transcription using the T7 high yield RNA synthesis kit (New England Biolabs) according the manufacturer’s directions. RNA was cleaned and concentrated (Zymo RNA Clean and Concentrator kit, ZYMO, USA) prior to injection. Primers were also tagged for fluorescent PCR and fragment analysis. Forward primers were tagged with an M13, and reverse primers were tagged with a PIG tail for fragment analysis. All oligonucleotides used as described above are listed below:

*Universal Ultramer*^22^: 5′ AAAAGCACCGACTCGGTGCCACTTTTTCAAGTTGATAACGGACTAGCCTTATTTTAACTTG CTATTTCTAGCTCTAAAAC-3′

*GR:* TAATACGACTCACTATAGGAATCGTCGCCAAAGATGGGTTTTAGAGCTAGAAATAGC

*GR-FW primer*: tgtaaaacgacggccagtAACAAACGAGCAACTGAGGG

*GR-RV primer*: gtgtcttTTAAGGTCTGCAATGCTGGC

*MR:* TAATACGACTCACTATAGGAAACTGTACCCTCTTTGTGTTTTAGAGCTAGAAATAGC

*MR-FW primer*: tgtaaaacgacggccagtGGCTTGTACATGAATGCTGCC

*MR-RV primer:* gtgtcttGGGCTCCCACTTGTTTTGGCC

*M13 Primer (FAM or HEX conjugated):* FAM/HEX-TGTAAAACGACGGCCAGT (100 mM)

*F0:* Embryos at the 1–2 cell stage were microinjected with 300 pg of Cas9 protein and 50 pg of sgRNA into the yolk and raised as described above. To assess the somatic activity of our target guide, we amplified the region using fluorescent PCR and used fragment analysis to separate the size of the fragments using capillary electrophoresis. Briefly, the fluorescent conjugated M13 primer will be incorporated during PCR amplification and when run on a genetic analyzer with known size standards, it will provide a signal that is reflective of the amplicon size^22,44,45^. Samples were prepared from single embryos by first extracting genomic DNA using the Extract-N-Amp kit (Sigma, USA). The resulting gDNA was diluted 10x and 1 μl was added to our PCR master mix (Taq DNA polymerase [1U/ml; Life Technologies 10342020], 1x Taq Buffer [Life Technologies], 0.2 µM primer mix [FW, RV + M13], 0.2 µM dNTPs, 1.5 mM MgCl_2_) to a final volume of 10 µl. The PCR reaction was run under the following conditions: 95 °C, 5 min; 35 cycles of 94 °C, 1 min, 57 °C, 1 min; 72 °C, 30 sec; followed by 72 °C, 5 min. Samples were then sent to the University of Calgary DNA Sequencing facility for fragment analysis on an Applied Biosystems 373XL genetic analyzer, where they were separated based on size using capillary electrophoresis. The size of each fragment was analyzed using Peak Scanner (Applied Biosystems, USA). Once somatic activity was confirmed, we outbred the injected fish to wildtype fish to determine germ-line transmission and reduce enrichment of off-target effects. Several mutant alleles were recovered for each gene; however, only one mutation for each gene was characterized and described here.

*F1*: Juvenile (2-month-old) F1 fish were fin-clipped from the caudal fin and genomic DNA was extracted as described above. Amplification of the target region with PCR was performed as described above (without the fluorescent primer). The resulting PCR product was cleaned-up using ExoSap (Thermo Fisher Scientific) as per the manufacturer’s instructions. Samples were sequenced by Sanger sequencing at the University of Calgary’s DNA sequencing facility. These heterozygous fish were bred as described previously^22^. The resulting progeny were F2.

*F2*: Juvenile (2-month-old) F2 fish were fin-clipped and gDNA was extracted. Fluorescent PCR and fragment analysis was performed for genotyping, and fish were subsequently sorted into WT, heterozygous and homozygous fish.

*F3*: The progeny (maternal-zygotic homozygous larvae) from F2 homozygous (zygotic) fish were used in all experiments. The use of maternal-zygotic mutants in this paper was designed to eliminate any contribution of the maternal glucocorticoid system to the ontogeny of the stress response.

### nr3c1 functional experiment

To determine whether the mutant nr3c1 homozygous fish (GR^−/−^) had a functional GR, 72 hpf WT and GR^−/−^ larvae were transferred to a 6-well plate (Sarstedt, USA). Each well contained 20 larvae and 4 ml of E3 embryo media. Larvae were exposed to either a vehicle (0.05% ethanol) or cortisol (5 μg/ml hydrocortisone; Sigma) for 24 h. 96 hpf larvae were euthanized in MS222 (0.3 g/L; Sigma), collected in pools of 10, all media removed and stored at −80 °C for later analysis of GR-responsive *11β-hsd2* transcript abundance. As there is no known target gene for MR characterized in fish yet, functional analysis via transcript abundance of a target gene of nr3c2 could not be completed.

### Stress Experiment

The zebrafish larval stress response was assessed in WT, GR^−/−^ and MR^−/−^ larvae (96 hpf) by subjecting fish to an acute swirling stressor^21^. *Setup:* Fish at 80 hpf were transferred to 50 ml Falcon tubes (10–20 fish/tube) containing 20 ml of E3 media and kept in a temperature (28.5 °C) and light controlled incubator overnight. There was a separate tube for each time point (4 tubes/genotype) and 10 larvae were pooled to obtain a sample size (n) of 1. This experiment was repeated on at least three different days (from different clutches) to ensure an appropriate sample size. *Stressor:* Fish at 100 hpf (time 0) were subjected to a swirling stressor; larvae were vortexed at 250 rpm for 1 min and allowed to recover. *Sampling:* The time 0 fish were sampled prior to the stressor and immediately euthanized with an overdose of MS222 (0.3 g/L). The remaining larvae post-stressor were sampled at 5, 10, and 30 min as described above. For cortisol quantification a sample size of one consists of pools of 10 larvae. Therefore, euthanized larvae were sampled as pools of 10 in a microcentrifuge tube, E3 media removed and stored at −80 °C for later analysis.

### Cortisol quantification

For cortisol analysis during development, pools of embryos and larvae were disrupted by sonication (3 s pulse, ~5x) in 10 μl/larva in 50 mM Tris buffer (pH 7.5) with a protease inhibitor cocktail (Roche Diagnostics, USA). Samples were centrifuged at 13,000xg for 1 min. The supernatant was removed and stored at −80 °C until use. Cortisol was quantified using an ELISA as previously described^23^.

### Western blotting

SDS-PAGE and Western blotting was performed as previously described^46^. Briefly, samples were homogenized in 50 mM Tris buffer (pH 7.5) with a protease inhibitor cocktail (Roche). The homogenate was sonicated (3 s pulse, ~5x) and centrifuged (13,000 xg for 2 min). The supernatant was removed, and the protein concentration was determined using the bicinchoninic acid (BCA) method using bovine serum albumin (BSA) as the standard. The homogenate was then diluted with Laemmli’s buffer (156.25 mM Tris, 50% glycerol, 5% SDS, 0.0625% bromophenol blue and 25% 2-mercaptoethanol). Samples were stored at −20 °C. Equal amounts of protein (40 μg) were separated on an 8% polyacrylamide gel and transferred to nitrocellulose membrane using a SemiDry transfer unit (BioRad). After transfer, membranes were blocked with a solution of powdered skim milk (5% w/v in TTBS (20 mM Tris, 300 mM NaCl, pH 7.5 with Tween 0.1%) containing 0.02% sodium azide). The primary antibodies included anti-trout GR antibody^47^ used at a dilution of 1:1000, and anti-zebrafish MR^48^ at 1:500 dilution. Antibodies were prepared in the blocking solution and membranes were incubated overnight at 4 °C. Membranes were then washed with TTBS (5 min, 3x) and incubated for 1 h with secondary antibody (1:3000 goat anti-rabbit IgG; Bio-Rad, 170–6515). Bands were detected with Clarity Western ECL substrate (BioRad, 170–5061). Molecular mass of the bands was confirmed using a low range molecular weight marker (FroggaBio, Canada), and the specificity of anti-GR was confirmed using rainbow trout liver homogenate. Equal loading was confirmed using a CY3 conjugated anti-β-actin (Sigma, C5838).

### Transcript abundance

Transcript levels of specific genes were measured by quantitative real-time PCR (qPCR). Total RNA was extracted from larvae using Ribozol reagent (VWR, Canada) according to the manufacturer’s instructions and quantified using a SpectraDrop Micro-Volume microplate (VersaMax, Molecular Devices, USA). One microgram of RNA was treated with DNase I (Thermo Scientific, USA) to remove genomic contamination prior to cDNA synthesis using the High Capacity cDNA Reverse Transcription Kit (Applied Biosystems, USA), according to the manufacturer’s protocols. Transcript levels were measured by qPCR in duplicate using gene-specific primers as described previously^23^. See^21^ for primer specific sequences and annealing temperatures.

### Behavioural Analysis

Light/Dark zebrafish larval behaviour was performed as described previously^28^. Briefly, 80 hpf larvae were transferred to clear 96-well plates with lids and allowed to acclimate overnight (96 hpf at time of measurement). Analyses were performed in a temperature-controlled room (28.5 °C). The movement of 96 hpf larvae was video captured and quantified using the Zebrabox infrared camera setup and tracking extension of the ZebraLab software system (Viewpoint Life Sciences, Canada). In all behavioural protocols, the animal colour was set to black and the background-subtracted detection threshold was set to 20. The integration period for movement data was set to 30 s. The light-dark protocol consisted of alternating periods of light and dark every 7.5 min (450 s). This 15 min light/dark cycle was repeated four times for a total of 60 min. The total distance moved (mm) was calculated every 30 s integration period and was a sum of distance covered during inactivity, small movement (2 mm/s–10 mm/s) and large movement (>10 mm/s).

### Statistics

Data are shown as mean ± SEM, and statistical comparisons analysed using Sigma Plot 13 (Systat Software, Inc). Ontogeny data were analysed using a two-way ANOVA (Holm-Sidak post hoc). All data were transformed to meet the assumptions of normality and equal variance. Untransformed data are shown in all figures. A significance level of p < 0.05 was used in all cases.
